# Lack of correlation between surface expression and currents in epileptogenic AB-calmodulin binding domain Kv7.2 potassium channel mutants

**DOI:** 10.1080/19336950.2018.1511512

**Published:** 2018-09-26

**Authors:** Alessandro Alaimo, Ainhoa Etxeberria, Juan Camilo Gómez-Posada, Carolina Gomis-Perez, Juncal Fernández-Orth, Covadonga Malo, Alvaro Villarroel

**Affiliations:** Instituto Biofisika, Consejo Superior de Investigaciones Científicas, CSIC, UPV/EHU, Leioa, Spain

**Keywords:** Calmodulin, channelopathy, epilepsy, KCNQ, Kv7, surface expression

## Abstract

Heteromers of Kv7.2/Kv7.3 subunits constitute the main substrate of the neuronal M-current that limits neuronal hyper-excitability and firing frequency. Calmodulin (CaM) binding is essential for surface expression of Kv7 channels, and disruption of this interaction leads to diseases ranging from mild epilepsy to early onset encephalopathy. In this study, we addressed the impact of a charge neutralizing mutation located at the periphery of helix B (K526N). We found that, CaM binding and surface expression was impaired, although current amplitude was not altered. Currents were reduced at a faster rate after activation of a voltage-dependent phosphatase, suggesting that phosphatidylinositol-4,5-bisphosphate (PIP_2_) binding was weaker. In contrast, a charge neutralizing mutation located at the periphery of helix A (R333Q) did not affect CaM binding, but impaired trafficking and led to a reduction in current amplitude. Taken together, these results suggest that disruption of CaM-dependent or CaM-independent trafficking of Kv7.2/Kv7.3 channels can lead to pathology regardless of the consequences on the macroscopic ionic flow through the channel.

## Introduction

**Figure 1.**
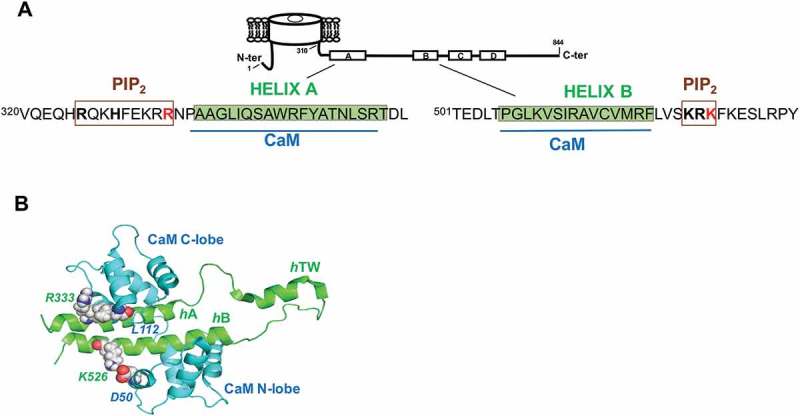
Kv7.2 Helices A-B architecture. (a), Cartoon representation of a Kv7.2 subunit. The amino acids corresponding to helices A and B are shadowed in green [6,26]. The CaM binding domains are indicated in blue, while putative residues of Helix A [28,29] and Helix B [30] implicated in the interaction with PIP_2_ are boxed in brown. The residues mutated R333 and K526 are in red. (b), Structure of the CaM/Kv7.2-*h*AB complex. Ribbon diagram of the complex between Kv7.2 AB helices and CaM [26]. The N-lobe and C-lobe of CaM are colored in blue and the helices A (*h*A) and B (*h*B) are in green. The Kv7.2 residues R333 and K526 and the putative interacting CaM aminoacids L112 and D50 are indicated. The figure was rendered using Pymol.

Kv7.2 and Kv7.3 heterotetramers (encoded by the *KCNQ2* and *KCNQ3* genes, respectively) are the main component of the neuronal M-current, a non-inactivating voltage dependent potassium current which controls neuronal excitability and firing frequency [1,2]. Consequently, mutations in these genes underlie genetic excitatory neuropathological conditions such as epilepsy or encephalopathy [2,3]. As many other potassium channels, Kv7 possess a long intracellular C-terminal region that plays a fundamental role in channel function and modulation in all the members of this family. It contains domains implicated in subunit assembly and diverse consensus motifs responsible for the interaction with other auxiliary proteins and lipids essential for the regulation of channel activity [4]. The calcium binding protein calmodulin (CaM) and the membrane phospholipid phosphatidylinositol-4,5-bisphosphate (PIP_2_) are unquestionably the most important (Figure 1(a)).

CaM, whose binding site is created by helices A and B in the C-terminus (Figure 1(a)), is required to produce functional Kv7.2 channels [5,6]. CaM affects not only PIP_2_ sensitivity [7,8], but also influences voltage sensitivity [7,9,10], regulation of the stability of the distal tetramerization domain [11], functional connection between this distal coiled-coil tetramerization domain and PIP_2_ modulation [12] and plays a role in the suppression of Kv7.2/Kv7.3 currents mediated by bradykinin [13,14]. Furthermore, CaM plays a crucial role in Kv7.2 channel trafficking [15], therefore regulating polarized axonal surface expression [16,17] and the intracellular transport of Kv7.2 proteins to the plasma membrane [18].

Mutations in the genes encoding human Kv7.2 give rise to benign familial neonatal epilepsy (BFNE), a dominantly inherited idiopathic epilepsy [1]. It was initially proposed that the reduction in current amplitude is the primary defect leading to disease, with a mere a 25–50% decrease of the M-current being sufficient for pathology [19–21]. Reduction in current levels can be the consequence of a myriad of processes, such as an increase in the degradation rate, reduction in PIP_2_ binding, impaired trafficking to the plasma membrane, or dysfunction in the biophysical properties of the channel; all of which have been proposed as responsible for the BFNE phenotype [15,18,22–25].

In this report, we examined two Kv7.2 BFNE associated missense mutations located in the periphery of the AB CaM-binding domain. The R333Q mutation is located adjacent to the contact site between the CaM C-lobe with helix A, whereas K526N is located next to the contact site of the N-lobe with helix B [26]; (Figure 1(a,b)). K526N was identified in an Italian family with two members affected by BFNE. Two other members also presented epileptic encephalopathy and mental retardation. The electrophysiological properties of both mutants have been previously analyzed [20,27], but the impact on CaM binding and the effect on channel trafficking and function remains unexplored. We have examined the consequences on CaM binding, exploring the effect on channel trafficking and function. Both mutants disrupted surface expression by different mechanism: whereas K526N compromised CaM binding, R333Q did not.

## Results

**Figure 2.**
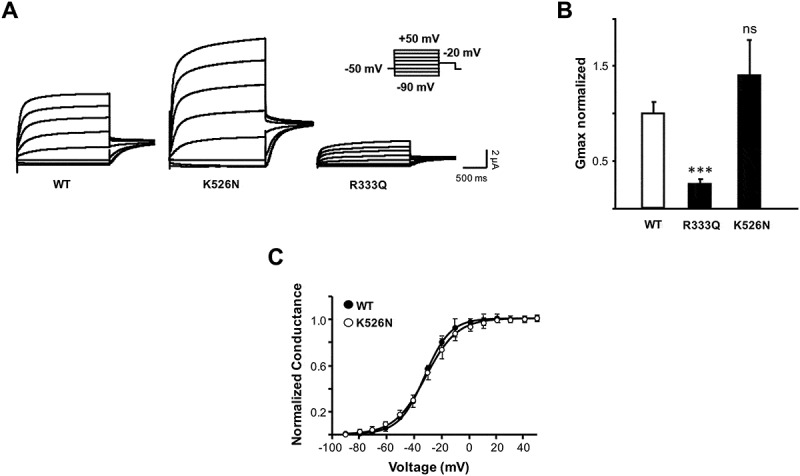
Characterization of BFNE-causing mutations in oocytes. (a), Representative current recordings from *Xenopus* oocytes injected in a 1:1 ratio with cRNAs for Kv7.3 and Kv7.2, or either the K526N or R333Q mutated Kv7.2 subunits. Inset: imposed voltage protocol. Each trace corresponds to the currents recorded in response to membrane depolarizations in 20 mV increments from −90 mV to + 50 mV (2 s duration), followed by a pulse to a constant voltage of −20 mV (1 s). (b), Normalized average maximal conductance of the indicated Kv7.2 subunits co-expressed with Kv7.3 (n ≥ 10 from 2 or more batches of oocytes). The following parameters were obtained after fitting a Boltzmann distribution to I-V relationships from tail currents measured at −20 mV: Kv7.2/Kv7.3: V1/2 = −41.7 ± 0.4, S = 9.4 ± 0.4; Kv7.2_R333Q_/Kv7.3: V1/2 = −39.1 ± 0.4, S = 8.8 ± 0.8; Kv7.2_K526N_/Kv7.3: V1/2 = −40.1 ± 0.26, S = 9.4 ± 0.5. Asterisks indicate values significantly different (*** = p < 0.001) versus WT-Kv7.2/Kv7.3. (c), Normalized conductance–voltage relationship of tail currents measured in 800 msec current traces at −20 mV for Kv7.3 channels co-expressed with WT Kv7.2 (*black circles*) or K526N (*white circles*). Boltzmann distributions were fitted to the data (continuous lines). Each point is the mean ± SEM of the data recorded in 10 or more oocytes.

The functional consequences of the K526N mutation were evaluated by co-expressing mutated Kv7.2 subunits with Kv7.3 subunits in *Xenopus* oocytes. Despite the functional properties observed may not be identical to those characterized in neurons, *Xenopus* oocytes remain one of the most standardized expression system used for the functional characterization of ion channels. In agreement with previous reports [27], K526N mutation did not cause a reduction in current amplitude (Figure 2). On the contrary, it tended to yield larger currents, but the differences with WT channels were not statistically significant.

When mapped in the NMR structure of the CaM complex, a potential interaction between K526 with a lateral helix of the CaM N-lobe was observed (Figure 1(b)). Interestingly, the topologically equivalent residue in helix A is the positively charged residue R333 that may interact with a lateral helix of the CaM C-lobe (Figure 1(b)). Therefore, we compared the consequences of the charge neutralization R333Q mutant. No significant changes on voltage dependence were observed for the heteromeric channels carrying Kv7.2 K526N (Figure 2(c)) or R333Q subunits (data no shown), whereas R333Q caused a large reduction in current (~80%), in accordance with previous reports [20]; (Figure 2(a,b)).

**Figure 3.**
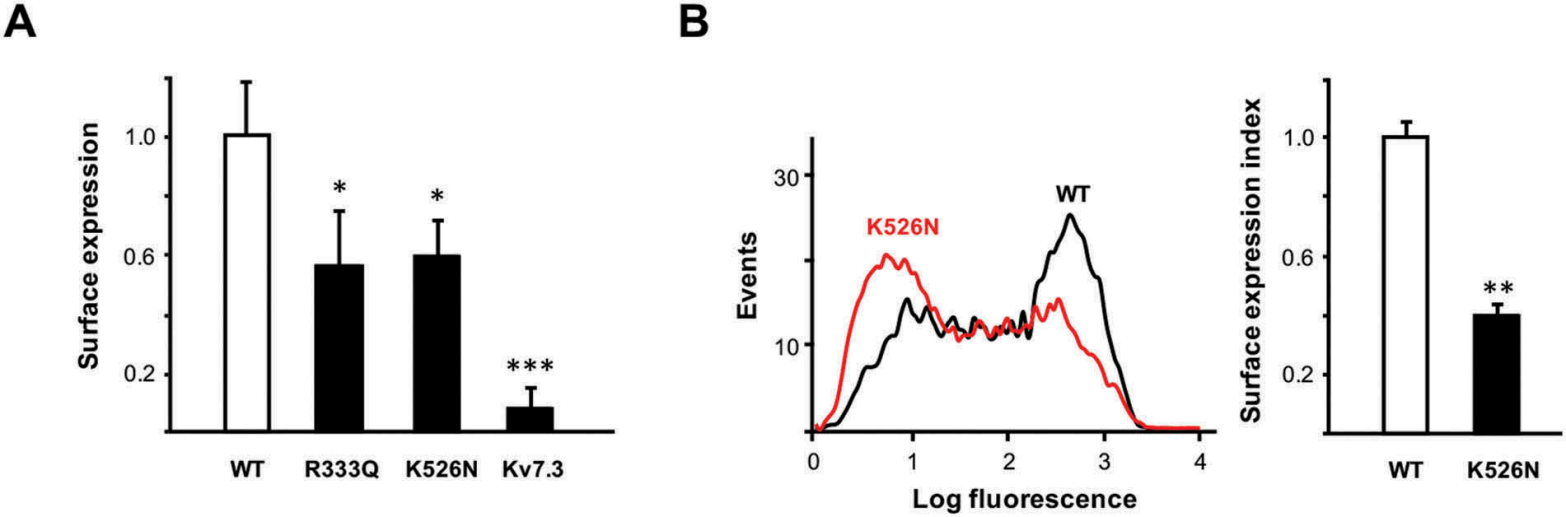
Surface expression is reduced in K526N and R333Q mutants. (a), Relative surface expression levels of Kv7.3-HA co-expressed with Kv7.2 subunits (n ≥ 11) in *Xenopus* oocytes. Kv7.3/Kv7.3-HA represents the negative control. The number of Kv7.3-HA containing channels in the oocyte membrane was quantified using a whole-oocyte chemiluminescence assay. The background of uninjected oocytes was subtracted, and the values given are the means ± SE normalized to values obtained from WT-Kv7.2/Kv7.3-HA channels from the same batch. Asterisks indicate values significantly different (* = p < 0.05; *** = p < 0.001) versus WT-Kv7.2/Kv7.3-HA. B *Left*, Representative flow cytometry histogram distribution of surface stained HEK293T cells expressing Tac-AB-CFP reporter construct carrying the indicated constructs. (b) *Right*, Plot of the surface expression index obtained from the normalized sum of the product of the number of events and fluorescence intensity from the flow cytometry histogram distribution (n = 4; ** significance at p < 0.01).

To investigate the trafficking of the channels to the plasma membrane, Kv7.3 subunits tagged with an HA epitope at an extracellular loop were used as reporter for surface expression in *Xenopus* oocytes [15,31,32]. Consistent with the reduction in current amplitude, R333Q mutant surface expression was diminished (~ 50%). Remarkably, although the K526N mutant did not diminish current, surface expression was reduced by 40% (Figure 3(a)). To confirm the impact of K526N in trafficking in mammalian cells, we fused the AB domain to the membrane protein Tac (interleukin-2 receptor α subunit) and monitored surface expression by flow cytometry in HEK293T cells as previously described [15,33,34]. The results confirmed that this mutation caused a reduction in surface expression (Figure 3(b)).

**Figure 4.**
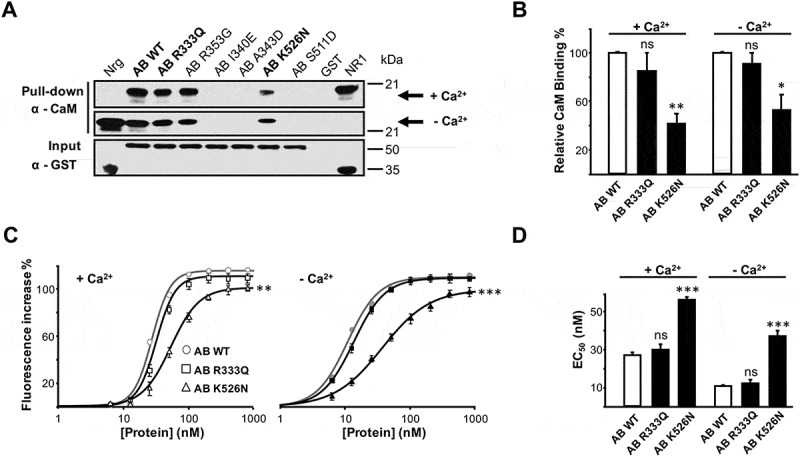
The K526N mutation impairs CaM binding. (a), Anti-CaM immunoblot after GST pull-down of GST-Kv7.2 (AB) with added CaM (10 μg) in the presence of Ca^2+^ (100 μM) or EGTA (5 mM). GST-neurogranin (Nrg, aa 1–78) and GST-NR1 (C0C1C2 C-terminal region of the NR1 receptor, aa 818–922) were used as positive controls to demonstrate interactions with apo-CaM and Ca^2+^-CaM, respectively. (b), Relative immunoblot signal of CaM. The data were obtained by WB from the following (n = 3; percent ± SEM): WT, 100.0 ± 1.0; R333Q, 85.2 ± 14.5; K526N, 41.4 ± 8.5, in the presence of Ca^2+^ and WT, 100.0 ± 0.8; R333Q, 91.1 ± 8.7; K526N, 52.9 ± 12.4, in the absence of Ca^2+^. Asterisks indicate values significantly different (* = p < 0.05; ** = p < 0.01) versus WT. (c), D-CaM (12.5 nM) fluorescence enhancement in the presence of Ca^2+^ (3.9 μM free Ca^2+^, *left*) or in the absence of Ca^2+^ (10 mM EGTA added, *right*) for the indicated recombinant GST-fusion protein concentrations. The lines are the result of fitting a Hill equation to the data. Asterisks indicate values of maximal fluorescence significantly different (** = p < 0.01; *** = p < 0.001). The data represent the means ± SEM from 3 or more independent experiments. (d), Plot of the apparent binding affinity (EC_50_) obtained from concentration-response curves as in C (D-CaM 12.5 nM). The apparent binding affinity was derived from 3 or more experiments. The EC_50_ (nM) values obtained were: WT, 27.1 ± 1.2; R333Q, 30.1 ± 2.8; K526N, 55.9 ± 1.5, in the presence of Ca^2+^ and WT, 11.0 ± 0.5; R333Q, 12.6 ± 1.3; K526N, 37.3 ± 2.4, in the absence of Ca^2+^ (n ≥ 3). The data for WT are from [36]. Asterisks indicate values significantly different (*** = p < 0.001) versus WT.

Some mutations linked to BFNE cause ER retention and a consequent reduction in surface expression due to disruption of CaM binding [15,18]. CaM binds the non-continuous domain formed by helix A and B of Kv7 channels [6,26]; (Figure 1(a)). Since both neutralizing mutations are adjacent to the interaction site (Figure 1(a,b)), we tested how CaM binding was affected. To investigate this, two *in vitro* assays were performed (Figure 4).

Firstly, the direct interaction between CaM and Kv7.2 helices A-B was assessed by *in vitr*o pull-down experiments using recombinant proteins (AB; Figure 4(a)). GST-Kv7.2 Helices A-B fusion protein carrying the K526N mutation was purified. Other mutations, (A343D, I340E in helix A and S511D in helix B), known to disrupt CaM binding [6], or to cause BFNE (R353G) [15,35], were used as negative controls, (Figure 4(a)). We also examined two CaM binding proteins fused to GST as positive controls for the interaction in the presence (the C-terminus of the NMDA receptor, NR1a) or absence (neurogranin) of Ca^2+^ [6,18]. Fusion proteins were immobilized on GSH-Sepharose beads and incubated with CaM, both in the presence and absence of Ca^2+^, and anti-CaM antibodies were then used in Western blot to detect the AB-CaM interaction (see “Materials and methods” section). Pull-down assays showed that in the presence of Ca^2+^ (Figure 4(a), *upper panel*), the A343D, I340E and S511D mutants did not interact with CaM above detectable levels [6,15,18] and that K526N exhibited a significantly weaker CaM signal than WT (42% lower signal intensity than WT; n = 3, Figure 4(b)). In contrast, the signal for the BFNE mutant R333Q was comparable to that of WT (Figure 4(a,b)). The mechanism leading to reduction in surface expression and consequent decrease in current amplitude for this mutant was not investigated further, whereas we concluded that it was not due to the disruption of CaM binding.

The results obtained after heavy Ca^2+^ chelation (10 mM EGTA, Figure 4(a), *middle panel*) were qualitatively similar to those observed in the presence of this cation. Accordingly, the semi-quantitative data obtained using this pull-down approach revealed that the K526N mutation significantly affected the interaction of CaM with Kv7.2 subunits, both in the presence and in the absence of Ca^2+^.

To further evaluate the impact on CaM binding, we monitored fluorescence changes of the dansyl group covalently attached to CaM (D-CaM). The low concentration and the GST tag help prevent protein aggregation, and, with the use of this very sensitive assay, quantitative data for the interaction was obtained [10,36,37]. Dose–response binding curves were constructed titrating D-CaM fluorescence after adding increasing concentrations of GST-fusion, in the absence (10 mM EGTA added; Figure 4(c) *right*) or in the presence of a physiologically relevant concentration of Ca^2+^ (3.9 μM free Ca^2+^; Figure 4(c) *left*). In the presence of Ca^2+^, an EC_50_ value of 55.9 ± 1.5 nM (n = 3) was obtained for the K526N mutant, that is about 2-fold lower affinity compared with WT (27.1 ± 1.2 nM; n = 5; Figure 4(d)). In the absence of Ca^2+^, the apparent affinity of K526N was 37.3 ± 2.4 nM (n = 3), a 3.5-fold lower affinity than for WT (11.0 ± 0.5 nM; n = 6) (Figure 4(d)).

**Figure 5.**
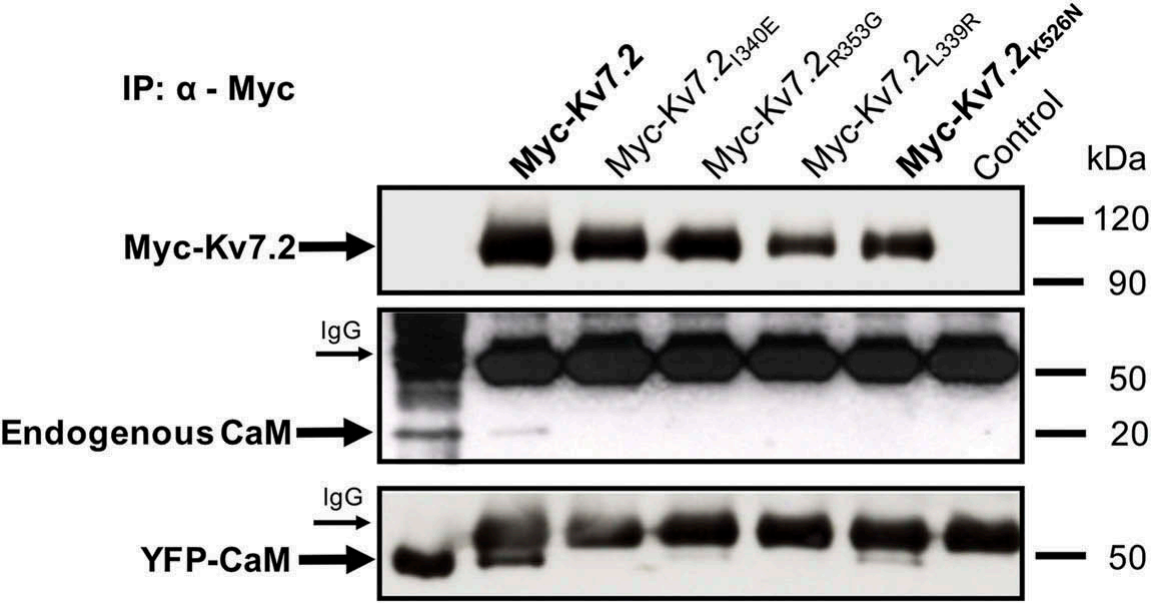
Co-immunoprecipitation experiments confirm altered interaction of CaM with the K526N channel. HEK293T cells were transfected with Myc-tagged Kv7.2 subunits, immunoprecipitated with an anti-Myc antibody, separated by SDS-PAGE, and analyzed by WB. The arrows indicate Kv7.2 subunits and immunoprecipitated endogenous CaM detected with anti-Myc (*top panel*) and anti-CaM antibodies (*middle panel*), respectively. The heavy chain of the anti-Myc antibody are also indicated with arrows. Cells were transfected with YFP-tagged CaM (YFP-CaM) to increase the amount of CaM in HEK293T, immunoprecipitated with an anti-Myc antibody, separated by SDS-PAGE, analyzed by WB and detected with an anti-CaM antibody (*bottom panel*). The densitometric quantification of CaM associated with the K526N mutant gave a value of 0.47, expressed as the ratio of the optical density of immunoprecipitated CaM/Kv7.2-K526N normalized to the CaM/WT-Kv7.2 value (n = 3) .

To extend the results to the complete channel, the intracellular N-terminal of Kv7.2 was tagged with 5xMyc and the construct was transiently transfected in HEK293T mammalian cells to test the interaction with endogenous CaM by co-immunoprecipitation (Figure 5). A faint ~ 20 kDa band was recognized by a CaM specific antibody in the immunoprecipitated obtained using antiserum to WT Kv7.2 subunits (Figure 5, *middle panel*). This result suggests that, probably, the levels of endogenous CaM in HEK293T cells may be limiting. To increase CaM levels in HEK293T, cells were co-transfected with YFP-tagged CaM (YFP-CaM), obtaining a substantial increase for CaM co-immunoprecipitated by WT Kv7.2 subunits (Figure 5, *bottom panel*). Importantly, there was a ~ 50% reduction in CaM signal when co-immunoprecipitated with K526N subunits (Figure 5, *bottom panel*). Thus, the results of the co-immunoprecipitation experiments confirmed those obtained using the pull-down and fluorescence assays, reinforcing the concept that the K526N mutation affects the interaction of Kv7.2 channels with CaM.

**Figure 6.**
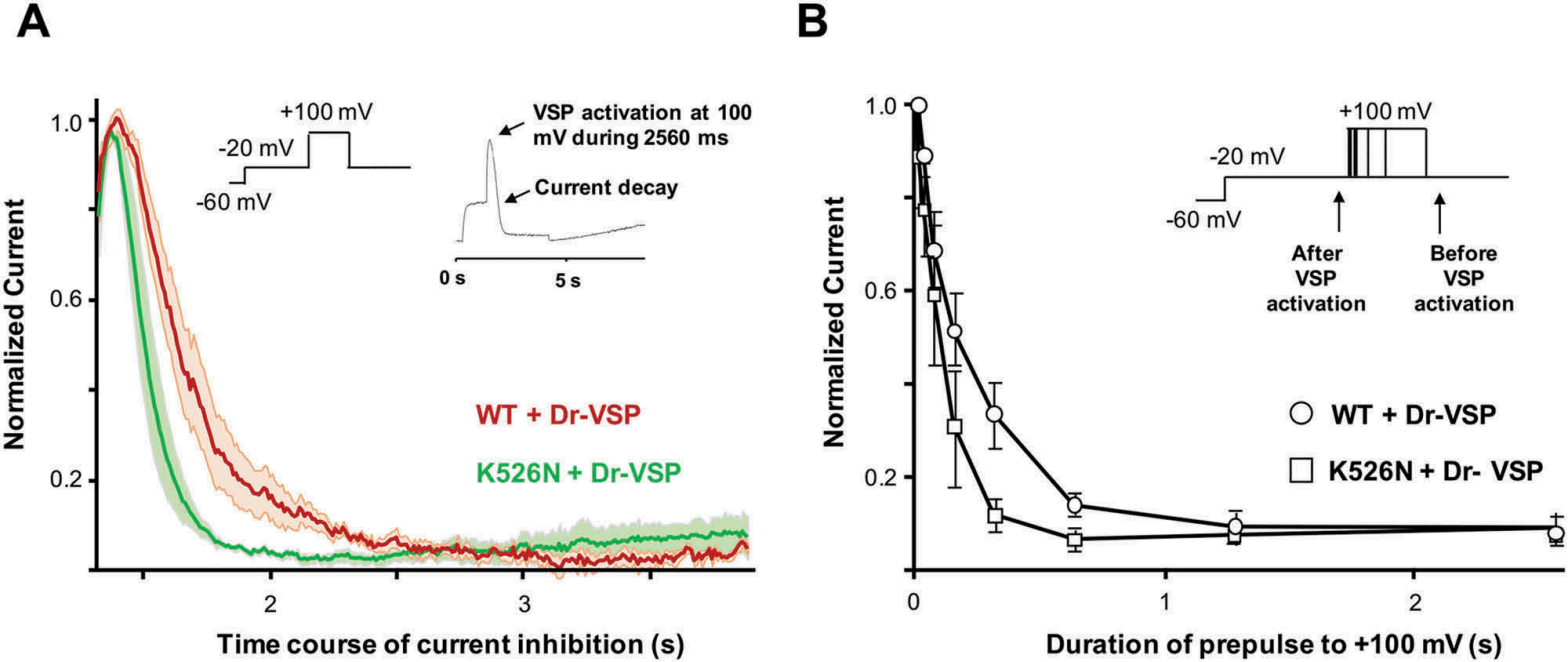
The K526N channel presents a reduced PIP_2_ affinity. (a), Averaged time course of the current decline during 2.560 ms of Dr-VSP activation at + 100 mV in cells transfected with WT + Dr-VSP (red, n = 6) or K526N + Dr-VSP (green, n = 6) subunits. The shadows represent the mean ± SEM. (b), Normalized current at 20 mV (after/before step to + 100 mV, see arrows in the inset) for different durations at + 100 mV. Each point represents the mean ± SEM for WT (n = 6) and K526N (n = 6) .

The reduction on CaM binding to the K526N mutant is consistent with the decreased surface expression observed, but the lack of impact on current amplitude suggests that a compensatory mechanism is playing a role. The ion channels macroscopic current is the result of the product of the number of functional channels, probability of the channel being open (P_open_), and single channel conductance. It seems unlikely that single channel conductance is affected by a residue that is not even in contact with the internal face of the pore, as revealed by the structure of the homologous Kv7.1 channel [38]. However, this residue is part of a cluster that affects the interaction with PIP_2_ in Kv7.1 channels [10], which should alter P_open_. To assess the impact of PIP_2_ sensitivity, the channel was co-expressed in HEK293T cells with a *Danio rerio* voltage-dependent phosphatase (Dr-VSP). This phosphatase, when activated by strong depolarizations (≥ 100 mV), reduces plasma membrane content of PIP_2_ and inhibits Kv7 channels [39,40]. Since the Kv7.2 channel *per se* does not inactivate, we measured the depolarization-induced current decay caused by the Dr-VSP-mediated PIP_2_ depletion. The rate of M-current decay is related to the affinity for PIP_2_, such as the faster the rate, the lower the affinity. We applied a simple protocol, described for the first time by Falkenburger and coworkers [40] starting with a depolarized step to −20 mV, a voltage in which the phosphatase is not activated, reveling the basal current level, then, a second pulse to + 100 mV with increasing duration, was applied activating Dr-VSP, and inhibiting the channel (Figure 6(b), inset). We compared currents at −20 mV before and after varying lengths of Dr-VSP activation to track the onset of the Dr-VSP effect. Figure 6(a) represent the averaged time course of the current decay during the maximum activation of the Dr-VSP (+ 100 mV during 2560 ms).

Contrary to our expectations, we found that the rate of current reduction was faster for the K526N mutant (Figure 6). We observed the same phenomena in Figure 6(b) where the average of inhibition of the current was measured at different pulse durations for of Dr-VSP activation. These results suggest that the K526N mutant channel has a lower PIP_2_ affinity compared to the WT.

## Discussion

In this study, using three different experimental approaches, we consistently demonstrated that the K526N mutation impairs binding to CaM (Figures 4 and 5). Our data indicate that there is a good correlation between the number of channels that reach the plasma membrane and the disruption/reduction of CaM binding caused by other pathological mutations [15,18]. As shown in Figure 3(a), surface expression of Kv7.2_K526N_/Kv7.3 channels were reduced by 40%, mirroring the decrease in CaM binding. Previous biotinylation assays in mammalian cells failed to resolve changes in surface expression [27], whereas the chemiluminescent assay employed in the present work revealed a clear reduction. To clarify this issue, we used another test based on Tac chimeras that confirmed the impairment on trafficking caused by this mutation in mammalian cells (Figure 3(b)). When mapped on the recently described Kv7.2AB/CaM complex, it appears that K526 could be making contacts with D50 located in EF2 of the CaM N-lobe [26], (Figure 1(b)). Aspartate 50 falls outside the groove between the two EF hands were the alpha helix of most target peptides accommodate. It precedes methionine 51, which is a major contributor to the interaction of the Ca^2+^ loaded N-lobe with many target peptides. However, in the absence of Ca^2+^ these residues are not making contacts with the target in many complexes [41]. Thus, our data suggest that the CaM_D50_/Kv7.2_K526_ interaction plays an important role in stabilizing the complex. The simplest explanation is that the disappearance of electrostatics interaction with CaM D50 by neutralizing the charge of the residue located at position 526 in Kv7.2 leads to a trafficking defect. Strikingly, and in agreement with previous reports [27], currents were not reduced, suggesting that there should be a compensatory mechanism at work.

Often, facilitation is manifested by a left shift of the voltage-current relationship, such as that caused by apo-CaM for Kv7.2 and Kv7.4 channels [7,9,42], or holo-CaM for Kv7.1 [10]. However, changes in these parameters were not observed (Kv7.2/Kv7.3 heteromers) or were in the opposite direction (Kv7.2 homomers) [27]. An increase in PIP_2_ binding affinity, which is an essential co-factor for Kv7 channel function, can also be discarded as the compensatory mechanism. A PIP_2_ binding site has been located at the tri-lysine cluster (_526_KKK_528_) of helix B in Kv7.1 [10], which is equivalent to the Kv7.2 _524_KRK_526_ basic cluster. The corresponding Kv7.1 K528 residue is not anticipated to interact with PIP_2_ according to docking and MS simulations, and the equivalent K528N mutant had little or no impact in the interaction with PIP_2_ *in vitro* [10]. Nevertheless, we found that currents decreased at a faster rate after activation of a voltage-dependent phosphatase for Kv7.2 channel carrying the mutation K526N, suggesting that this residue contributes in shaping the poly-basic cluster for its interaction with PIP_2_ in Kv7.2 channels (Figure 6).

Thus, all the properties affected by the K526N mutation analyzed, CaM binding, trafficking, voltage- and PIP_2_-dependency, should result in smaller currents, yet the current amplitude was not affected. Some compensatory mechanisms should be considered. An increase in single channel conductance would require a long-distance influence from this intracellular residue with the pore region that is difficult to rationalize in the Kv7.1 cryoEM structure [38]. Other possibilities to consider are an increase in the probability of the channel being open, or the unmasking of silent channels at the membrane [43].

The data obtained for the R333Q mutant were indistinguishable to those obtained with WT, confirming that this mutation did not affect the Kv7.2-CaM interaction (Figure 4(c,d)). These results are totally in line with the pull-down data and suggest that the K526N mutation impairs binding of Kv7.2 to CaM, both in the presence and in the absence of Ca^2+^, whereas R333Q does not affect the interaction of CaM with the binding domain (Figure 4(a,b)).

Thus, we have unveiled three consequences of the K526N mutation (impaired CaM binding, reduced surface expression, and increased PIP_2_-dependency) that should lead to reduction in current amplitude, yet the current levels were similar to that of WT channels. In contrast to K526N, the charge neutralizing mutation R333Q located at the periphery of helix A did not affect CaM binding. Nevertheless, it led to a reduction in surface expression accompanied with a decrease in current amplitude. Accordingly, contrary to the helix B mutation, there does not appear to be a compensatory mechanism that counteracts the reduction on the number of channels at the membrane.

The pathological mechanisms leading to disease remains unresolved. Surface expression impairment is a common feature for both mutants, which should interfere with targeting of these channels to the axon initial segment [16,44], which is essential for integration of signals and control of neuronal excitability [45]. Our results reveal that mutations in the AB/CaM complex can impair trafficking by CaM-dependent and – independent mechanisms. CaM has a dual effect on the Kv7.2/7.3 channels physiology: it participates in the traffic of the channels to the plasma membrane [15] and inhibits the M-current by monitoring the Ca^2+^ increase and PIP_2_ decrease produced by the activation of bradykinin receptors in sympathetic neurons [13,46]. It is expected that the increased PIP_2_ sensitivity and reduced CaM binding should change how neurons integrate modulatory signals based on this lipid or Ca^2+^. It will be of interest to address how Ca^2+^-mediated regulation contributes to the complex phenotype observed for the patients carrying the K526N mutation.

## Materials and methods

### Molecular biology

The human Kv7.2 (Y15065) and Kv7.3 (NM004519) cDNAs were a generous gift of Thomas J. Jentsch (Leibniz-Institut für Molekulare Pharmakologie, Berlin, Germany) and all point mutations and epitope insertions in the Kv7 subunits were constructed by PCR-based mutagenesis. The cDNA encoding rat CaM were provided by John P. Adelman (Vollum Institute, Oregon Health Sciences University, Portland, OR, USA). For surface expression experiments, the Kv7.3 subunit was tagged with a hemagglutinin (HA) epitope in the extracellular loop that connects transmembrane domains S1 and S2. The Tac-AB-CFP constructs were generated using the Tac receptor provided by Dr. Steve Standley (Laboratory of Neurochemistry, NIDCD, NIH, Bethesda, MD, USA), which was cloned into a modified version of the expression vector pEGFP (Clontech), where eGFP has been replaced for mCFP. The electrophysiological properties recorded in *Xenopus* oocytes of tagged Kv7.3 constructs were indistinguishable from the nontagged subunits. Dr-VSP-IRES-GFP (Dr-VSP) from zebrafish (*Danio rerio*) was obtained by Y. Okamura (Osaka University, Osaka, Japan).

### Cell culture and transfection

HEK293T cells were maintained in Dulbecco’s modified Eagle’s medium (DMEM) supplemented with 10% FBS at 37°C in 5% CO_2_. Cells were transiently transfected with cDNAs using calcium phosphate.

### Electrophysiology

*Xenopus* oocytes preparation and standard two-electrode voltage clamp were performed as previously described [31,47]. Stage V or VI oocytes were defolliculated with 1 mg/ml collagenase (C9891; Sigma) in Ca^2+^-free OR2 (5 mM HEPES, 82.5 mM NaCl, 2.5 mM KCl and 1 mM MgCl_2_, pH 7.5), and then were transferred to a Ca^2+^-containing solution ND96 (5 mM HEPES, 96 mM NaCl, 2 mM KCl, 1.8 mM CaCl_2_, 1 mM MgCl_2_, pH 7.5). The oocytes were injected with 50 nl of Kv7.2 or Kv7.3 cRNA solution, containing 10 ng of Kv7.2 (WT or mutated) or Kv7.3 cRNA or 10 ng of a 1:1 mixture in coexpression experiments. Three days after injection, whole-cell currents were recorded in oocytes at room temperature with a two-electrode voltage clamp using a virtual-ground Geneclamp 500B amplifier (Axon Instruments). Borosilicate electrodes were filled with 3 M KCl and presented resistances of 1 MΩ. The oocytes were perfused uninterruptedly in Xenopus saline solution: 5 mM HEPES, 100 mM NaCl, 2.5 mM KCl, 1 mM MgCl_2_ and 2 MnCl_2_, pH 7.5. Data were acquired at a sampling rate of 1 KHz and filtered at 100 Hz. Voltage-step protocols and current analysis were achieved with pCLAMP 8.2 software (Axon Instruments).

Current values represent the average (± S.E.) of the maximal conductance obtained from the fit of the g–V relationship to a Boltzmann equation:

$${\rm{y = }}{{\rm{G}}_{{\rm{max}}}}{\rm{/[1 + exp(}}\left({{{\rm{V}}_{{\rm{1/2}}}}{\rm{ - }}{{\rm{V}}_{\rm{m}}}} \right){\rm{/slope}}$$

where G_max_ = *I*_max_/(V_step_ – V_rev_), *I*_max_ is the maximal current, V_rev_ is the reversal potential (−87.3 ± 0.34 mV; *n* = 387; in our recording conditions), y is the tail current amplitude in an 800 ms voltage step to −20 mV (V_step_) elicited after 800 ms depolarizing voltage pulses from −120 to + 50 mV in 10 mV increments (V_m_) evoked every 20 s, and V_1/2_ is the membrane potential at which the current is half *I*_max_. To obtain the absolute amplitude, the tail current values were subtracted from the amplitude of the tail current preceded by a voltage step to −120 mV. The G_max_ value was normalized to the averaged G_max_ obtained the same day using ten or more oocytes expressing Kv7.2/Kv7.3 heteromers.

HEK293T cells were used to perform the experiments with Dr-VSP as described [39,40]. Briefly, Dr-VSP was activated by a 200 ms, jump to + 100 mV, and then the voltage was returned to the holding potential of −20 mV. Jumps to −110 mV were applied to close the channels. Upon returning to the holding potential, an instantaneous current jump (corresponding to leak current) followed by a slowly developing outward relaxation (corresponding to the opening of the M-channels) was recorded. The size of the outward relaxation before and after the + 100 mV jump was used to estimate the effect of Dr-VSP activation on M-current size [48,49]. The data were acquired and analyzed using pCLAMP software (version 8.2), normalized in Excel (Microsoft Corp.) and plotted in Sigmaplot 12 (SPSS Corp.).

### Surface expression

We followed the method based on luminescence described by Schwake and coworkers using the Kv7.3 subunit tagged with a hemagglutinin (HA) epitope (YPYDVPDYA) in the extracellular loop that connects transmembrane domains S1 and S2 [31,32]. Oocytes were injected with 20 ng of a 1:1 cRNA mixture that always included 10 ng of Kv7.3-HA. After three days at 19°C, the oocytes were placed in ND96 with 1% BSA (ND96/BSA) at 4°C for 30 min to block unspecific binding. Afterward, the oocytes were incubated in ND96/BSA for 60 min at 4°C with 1ug/ml of rat monoclonal anti-HA antibody (3F10; Roche Diagnostics), washed with ND96/BSA, and incubated in ND96/BSA with HRP-coupled secondary antibody for 60 min (goat anti-rat antigen binding fragments; Jackson ImmunoResearch). Finally, the oocytes were washed thoroughly with ND96/BSA and then with ND96 alone to eliminate the background signal that BSA produces. Individual oocytes were placed in 50 ul Power signal ELISA solution (Pierce), and chemiluminescence was quantified in a Sirius luminometer (Berthol).

### Flow cytometry

HEK293T cells grown in T25 Flasks were transiently transfected with Tac-Kv7.2 AB-mCFP by the calcium phosphate method. After 36 h, cells were detached, transferred to 1.5 ml Eppendorf tubes, washed and resuspended in 700 μl PBS. Cells were fixed for 20 min at room temperature with 3% paraformaldehyde (Fluka) and washed with PBS. After pre-incubation with 5% BSA (Sigma) for 30 min, cells were labeled for 1 h at room temperature with an antibody recognizing a Tac extracellular epitope, washed three times with PBS, and incubated for 1 h at room temperature with an Alexa-Fluor-488- conjugated anti-mouse-IgG secondary antibody. Cells were washed four times and resuspended in PBS before analysis on a Gallios flow cytometer (775014, Beckman Coulter). Data were collected from at least 10,000 healthy cells with emission intensities above the background level determined using untransfected HEK293T cells. Histograms normalized to 10,000 events were visualized using WinMDI 2.9 software, analyzed in Excel and represented using SigmaPlot.

### Immunoprecipitation

HEK293T cells were solubilized for 30 min at 4°C 24 h after transfection in IP buffer (50 mM Tris-HCl, 150 mM NaCl, 1% Triton X-100, 2 mM EDTA, 5 mM EGTA and protease inhibitors, pH 7.5). The nuclei were pelleted at 500 g for 3 min, followed by centrifugation at 11,000 g for 20 min. Lysates were precleared with 40 μl of equilibrated Protein A sepharose beads (Sigma P3391) for 1 h at 4°C. Anti-myc antibodies were immobilized with 40 μl of equilibrated Protein A beads O/N at 4°C and washed twice with IP buffer. Precleared lysates were incubated with Protein A-anti-myc for 4 h at 4°C and, after 4 washes with IP buffer, immunoprecipitated proteins were released by heating at 90°C 5 min in SDS sample buffer.

### Protein production

The G310–L548 region of Kv7.2 (WT, R333Q, K526N and the other mutants), neurogranin (aa 1–78, NP006167), and the C terminus of the NR1a NMDA receptor (aa 818–922; NP015566) were subcloned into the pGEX-3X vector (GE Healthcare). The expression of these GST fusion proteins in BL21 DE3 strain, the purification and the post-purification quality check of the proteins have been described elsewhere [18,50]. Recombinant rat brain calmodulin was produced in BL21 DE3 bacteria and purified as described [6].

### GST pull-down assay

Purified GST fusion proteins immobilized on GSH-Sepharose 4B beads (GE Healthcare) were equilibrated in pull-down buffer (25 mM Hepes, 120 mM KCl, 5 mM NaCl, pH 7.5) with either 100 μM CaCl_2_ or 5 mM EGTA. Calmodulin (10 μg) was added to the beads and incubated for 45 min at room temperature. After three washes, the proteins were recovered, separated by 15% SDS-PAGE, and transferred to Immobilon-P PVDF membrane (Merck Millipore) for Western blotting. The membrane was blocked with 5% nonfat milk in TBS-T (10 mM Tris-HCl, 150 mM NaCl, 0.1% Tween 20, pH 7.5), incubated with the primary antibody (monoclonal anti-CaM 05–173 from Merck Millipore, diluted 1:2000 in blocking buffer) O/N at 4°C, washed, and incubated with horseradish peroxidase-conjugated goat anti-mouse IgG secondary antibody (Bio-Rad) diluted 1:5000 in blocking buffer. Antibody binding was detected using enhanced chemiluminiscence and ECL hyperfilm (GE Healthcare). GST-fusion proteins (input) were identified using an Anti-GST–Peroxidase Conjugate antibody (dilution 1:5000; A7340 Sigma). ImageJ software (Version WCIF, National Institutes of Health, USA) was used to quantify band intensities and calculate the relative CaM binding % (n = 3).

### Fluorescence spectroscopy

CaM dansylation, sample and buffers preparation and fluorescent measurements using dansyl-CaM (D-CaM), were performed as described previously [11,18].

### Statistical analysis

Data were analyzed using unpaired t-test (GraphPad Prism 6, GraphPad Software, Inc.). Values of P < 0.05 were considered statistically significant. *P < 0.05; **P < 0.01; ***P < 0.001. The mean ± s.d. of three or more independent experiments is reported.
